# Functional constraints on HIV-1 capsid: their impacts on the viral immune escape potency

**DOI:** 10.3389/fmicb.2012.00369

**Published:** 2012-10-17

**Authors:** Taichiro Takemura, Tsutomu Murakami

**Affiliations:** AIDS Research Center, National Institute of Infectious DiseasesTokyo, Japan

**Keywords:** HIV-1, capsid, host factor, immune response, functional constraints

## Abstract

In mature HIV-1 particles, viral capsid (CA) proteins form the conical core structure that encapsidates two copies of the viral RNA genome. After fusion of the viral envelope and cellular membranes, the CA core enters into the cytoplasm of the target cells. CA proteins then interact with a variety of viral other protein as well as host factors, which may either support or inhibit replication of the virus. Recent studies have revealed that CA proteins are important not only for the uncoating step but also for the later nuclear import step. Identification of proteins that interact with CA to fulfill these functions is, therefore, important for understanding the unknown HIV-1 replication machinery. CA proteins can also be targets of the host immune response. Notably, some HLA-restricted cytotoxic T-lymphocyte (CTL) responses that recognize CA functional regions can greatly contribute to delay in AIDS progression. The multi-functionality of the CA protein may limit the flexible virus evolution and reduce the possibility of an escape mutant arising. The presence of many functional regions in CA protein may make it a potential target for effective therapies.

## Introduction

The HIV-1 *gag* gene encodes the Gag protein, major structural component of virus particles (Vogt, 1997; Scarlata and Carter, 2003; Engelman and Cherepanov, 2012). The Gag protein consists of six functionally different proteins. Capsid (CA) is the largest component of Gag protein, and forms core structure of the mature HIV-1 particle. Recent studies have revealed that the CA protein has multiple roles in the virus-host interaction at the cellular or individual levels. In this mini-review, we are summarizing the interaction of the CA and host cellular proteins such as cyclophilin A (CypA), Nuclear pore proteins (Nups), TRIM5alpha, and/or host immune response.

## HIV-1 capsid proteins constitute the viral core structure

**Figure 1:**
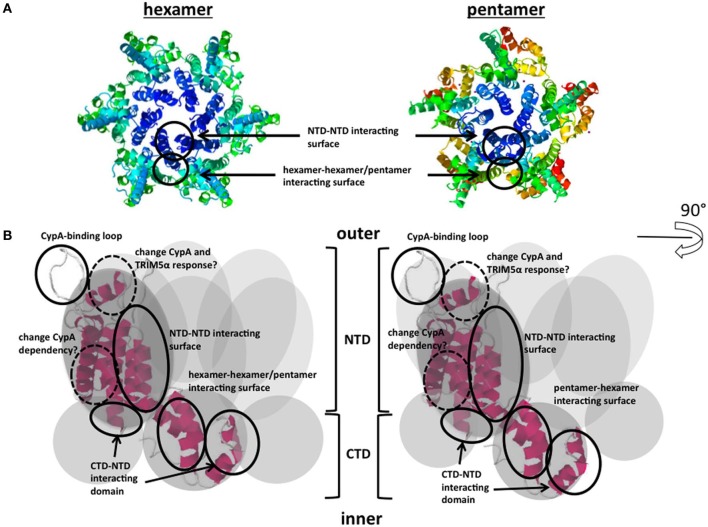
**CA proteins form hexamers and pentamers and schematic model of CA interacting domains. (A)** Hexameric/pentameric models proposed from structural analysis of the HIV-1 core. Each core comprises approximately 250 hexamers and exactly 12 pentamers. The accession numbers of protein Databases are 3H47, 3P05. **(B)** Functional surfaces in the CA protein. The gray circles indicate monomers of CA. NTD-NTD or CTD-NTD interacting surfaces, CypA-binding loops, and hexamer-hexamer/pentamer interacting surfaces are shown. CA; viral capsid; NTD; N-terminal domain, CTD; C-terminal domain. The accession numbers of protein Database are 3DIK. The detailed map including each component is shown in Figure 2.

The HIV-1 Gag proteins are synthesized as Pr55Gag polyprotein in cytoplasm of the virus-producing cell, and are then translocated to the plasma membrane. Subsequently, they are co-assembled into virus particles, which bud and are then released from the plasma membrane. Right after the budding and release step from the virus producing cells, virus particles undergo a process of maturation (Morikawa, 2003). The viral protease (PR) cleaves the Gag polyprotein into six proteins: matrix (MA), CA, nucleocapsid (NC), p6, p2, and p1. Virus morphology dramatically changes as a result of the maturation process. In the mature virus particles, CA proteins form a conical core structure encapsidating two copies of the viral RNA genome associated by NC proteins. The CA protein has N-terminal and C-terminal domains (NTD and CTD, respectively), and the short flexible linker connects between the two regions (Gitti et al., 1996; Momany et al., 1996; Gamble et al., 1997). The CA proteins assemble into the small units containing five or six monomers (Figure 1A) (Li et al., 2000; Pornillos et al., 2009, 2011; Yeager, 2011). The core structure is composed of approximately 250 units of hexamers, and 12 units of pentamers at the both conical ends.

The mature virus particles can then infect to the new target cells. The binding of the Env and receptors/co-receptors leads to fusion of the viral envelope and cellular membranes, and subsequently, the CA core enters the cytoplasm of the target cell. The CA core interacts with a variety of cellular proteins at this step (Mascarenhas and Musier-Forsyth, 2009). Before penetration into new target cells, the viral core should be stable to protect the viral RNA genome from the outer environment (Koh et al., 2000). However, after penetration, the core must be destabilized to uncoat and release the viral genome at the correct time for replication. Although spatial and temporal regulation of the uncoating process is not yet well understood, this process presumably depends on the interaction of CA with several host factors. The identification of such host proteins is, therefore, likely to be essential for understanding the HIV-1 replication cycle.

## Interaction of CA and host factors

### Cyclophilin A

**Figure 2:**
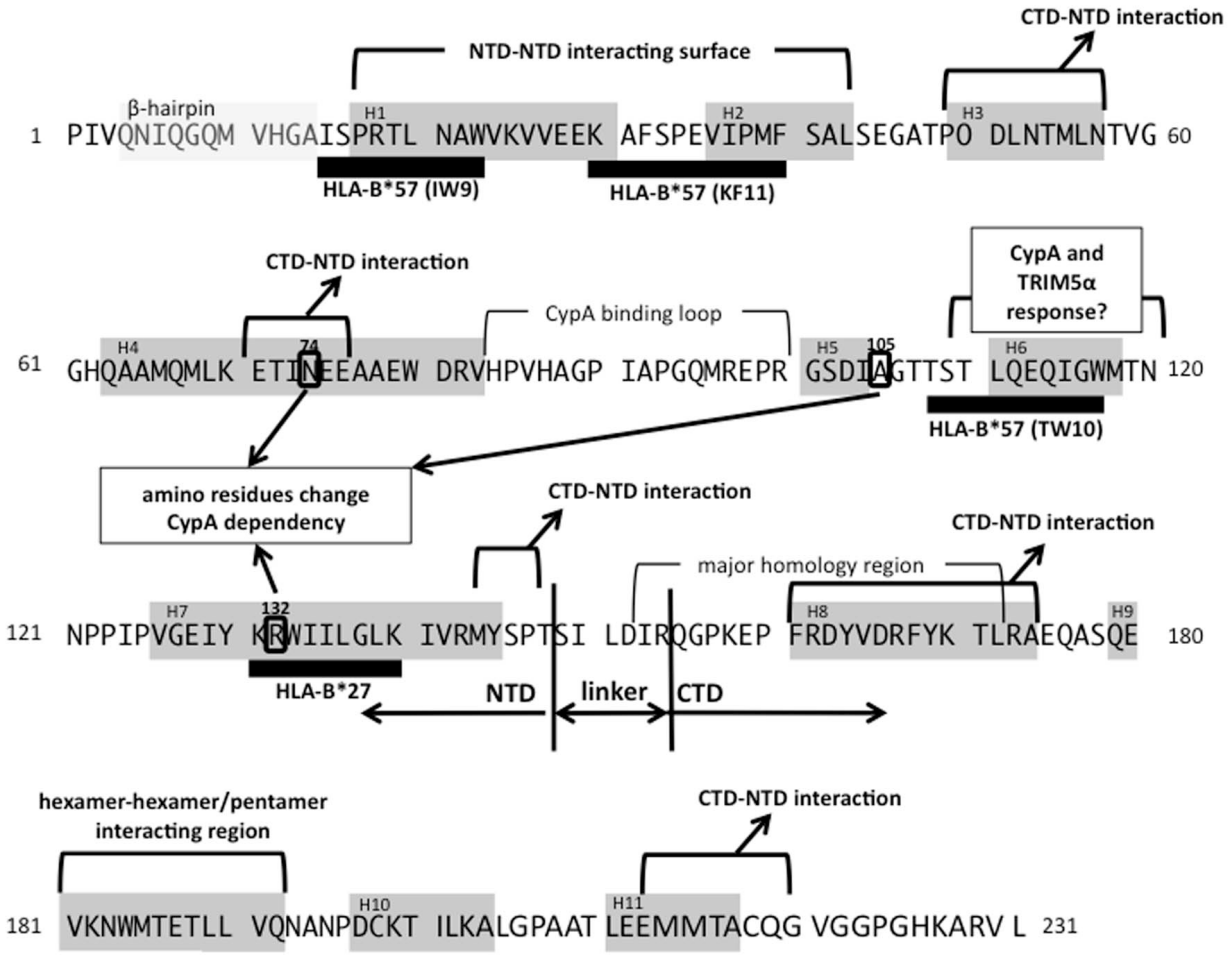
**Mapping of CA functional domains and two major protective CTL alleles.** Gray boxes show β-hairpin (amino acids 4–14) or helix structures (H1–H11). Thick black bars indicate the CTL epitopes restricted by each allele (Llano et al., 2009). The NTD-NTD, CTD-NTD, or hexamer-hexamer/pentamer interacting regions are indicated. Three amino residues (N74, A105, and R132), which are reported to change CypA dependency, are shown in circles (Schneidewind et al., 2007; Yang and Aiken, 2007; Ambrose et al., 2012). The reference amino acids sequences are from HXB2 (accession number; AB50258.1).

Cyclophilin A (CypA) is a host cellular protein that carries peptidyl-prolyl cis-trans isomerase (PPIase) activity and is abundantly expressed in various types of cell, including T-lymphocytes. CypA incorporates into HIV-1 particles via interaction with pr55Gag protein (Luban et al., 1993; Franke et al., 1994; Luban, 1996), binding at a site in the CA protein NTD, termed the CypA-binding loop (Figures 1B and 2). The CypA-binding loop is a proline-rich loop located between helices 4 and 5 in the CA protein, with the proline residue at position 90 considered to be the most important amino acid for CypA-binding (Grättinger et al., 1999). Interference in CA-CypA-binding reduces HIV-1 infection efficiency (Franke et al., 1994; Luban, 1996). Initial studies to determine the role of CypA in HIV-1 infection focused on CypA incorporated into virions, but later studies showed that the target cellular CypA is more important for HIV-1 infection (Sokolskaja et al., 2004; Hatziioannou et al., 2005). The exact role of CypA in the HIV-1 replication is still unclear and remains a subject of debate (Luban, 2007; Mascarenhas and Musier-Forsyth, 2009). One hypothesis is that the proline isomerase activity induces a conformational change in the core structure, which results in its destabilization and efficient viral uncoating in the target cell cytoplasm. Interestingly, CypA is required for the replication HIV-1 group M virus, but not always necessary for the HIV-1 group O or other simian immunodefficiency virus (SIV) replication (Braaten et al., 1996). Besides, Takeuchi et al. (2012) reported the opposing response to the CypA in HIV or SIV infection in different host cell species. Identification of the molecular basis of CypA-dependent HIV-1 infection may also contribute to understand the evolution of the HIV-1.

### Nup358 and other factors supporting nuclear import

The lentiviruses, including HIV-1, are able to infect non-dividing cells such as macrophages. The viral genome, consisting the pre-integration complex (PIC), must move into the nucleus of the target cell even in the non-dividing state, but molecular mechanism of the nuclear import step has yet to be determined. Numerous studies have examined the requirements for specific cellular proteins during HIV-1 nuclear import, including nuclear transport proteins such as some importin family proteins and Nups. It has been proposed that the PIC goes through the nuclear pore on the nuclear membrane (De Iaco and Luban, 2011). One of the Nups, Nup358 (also known as RanBP2 or RAN binding protein 2), is a cellular co-factor for the HIV-1 infection at the PIC nuclear import step (Hutten et al., 2009; Zhang et al., 2010; Ocwieja et al., 2011; Schaller et al., 2011). Nup358 is a relatively large protein (358 kDa) located on the cytoplasmic surface of the nuclear pore. Nup358 controls the cell cycle, nuclear export, and transportin-dependent nuclear import. Interestingly, Nup358 has a cyclophilin-like domain on its C-terminal end. Schaller et al. (2011) revealed that Nup358 directly binds to HIV-1 CA protein, with the viral acceptor being the CypA-binding loop described above. The knockdown of Nup358 in target cells impairs HIV-1 infection at the nuclear import step, and the migration of the PIC through the nuclear pore depends on interaction of Nup358 and CA (Schaller et al., 2011). Although the direct interaction of CA and Nup358 has now been identified, it is still unclear how Nup358 supports HIV-1 nuclear import. Furthermore, transportin-SR2 (TNPO3) was identified as a HIV-1 co-factor that supports the nuclear import step by a series of genome-wide siRNA screens, and TNPO3 interacts with CA (Brass et al., 2008; Krishnan et al., 2010; Lee et al., 2010; De Iaco and Luban, 2011). These observations suggest that the functional analysis of CA should be expanded to include nuclear import steps. Also, a recent study proposed a model in which reverse transcription and uncoating processes are regulated by each other or occurring at almost same time, at the perinuclear location in the target cells (Hulme et al., 2011). Further analysis into the role of CA in these steps, and their interactions with other host proteins including Nups should help to clarify the molecular mechanism of HIV-1 replication.

### TRIM5alpha

HIV-1 infection is strikingly restricted in the non-human primate cells. It had been predicted that the CA targeting dominant-acting inhibitory factor(s) expressed in these cells (Kootstra et al., 2003; Goff, 2004; Towers, 2007; Luban, 2012). Stremlau et al. (2004) identified TRIM5alpha as a species-specific HIV-1 restriction factor from the cDNA library of rhesus monkey cells. The other ortholog of TRIM5 gene (known as TRIM-Cyp), which restricts HIV-1 infection, was also identified from owl monkey cells (Nisole et al., 2004; Sayah et al., 2004). TRIM5alpha is a member of the tripartite motif-containing protein family, and the N-teminal domain has E3 ubiquitin ligase activity (Diaz-Griffero et al., 2006; Stremlau et al., 2006; Luban, 2012). Since the TRIM-Cyp carries a CypA-like domain in the C-terminal, it can targets the HIV-1 CA via the CypA-binding loop (Sayah et al., 2004). Although, the C-terminal domain of TRIM5alpha also has a function to recognize CA, its mechanism had not been clarified despite of the numerous studies. Ganser-Pornillos et al. (2011) proposed the TRIM5alpha lattice model, in which a spontaneously formed cellular TRIM5alpha hexameric lattice recognizes a surface of the incoming CA core. The detailed mechanism of TRIM5alpha- or TRIM-Cyp-mediated HIV-1 restriction after recognition of the core structure is still unclear. Two distinct restriction stages have been observed in both TRIM5alpha- or TRIM-Cyp-mediated HIV-1 restriction (Anderson et al., 2006; Yap et al., 2006). The unknown host factor may contribute to their restriction mechanism.

## CA protein and the host immune response

### Can the CA activate host innate immune sensor?

Manel et al. (2010) reported that the interaction of CypA and newly synthesized Gag proteins induces the type I interferon response to activate T-cells. They showed that the CypA-Gag interaction in dendritic cells activates the IRF3 pathway. This was the first report of an interaction between HIV-1 Gag (or CA) and CypA playing a role in inducing the host innate immune response. More recently, Pertel et al. (2011) showed that the interaction of host cellular TRIM5 and CA stimulates AP-1 and NF-κB signaling via the TAK-1 (also known as MAP3K7) pathway. The proposed mechanism for this immune sensor is rather complicated. In the presence of the heterodimeric E2 ubiquitin-conjugating enzyme, UBC13/UEV1A, TRIM5alpha catalyzes the synthesis of unattached K63-linked ubiquitin chains. The free K63-linked ubiquitin chain activates the TAK1 kinase complex, and the TAK-1-mediated signal then activates the inflammatory cytokine transcription via the NF-κB and AP-1 pathway. These two findings suggest that the Gag protein or core structure may be recognized by the TRIM5alpha or CypA that is acting as a pathogen recognition receptor for the host innate immune response. Further work in this area may contribute to the development of new therapeutic strategies utilizing the host immune response.

### Major protective cytotoxic T-Lymphocyte (CTL) alleles targeting CA protein

The host cytotoxic T-lymphocyte (CTL) response is a major effector to control HIV-1 replication *in vivo* (Borrow et al., 1994; Goulder and Watkins, 2008). CD8+ CTLs recognize the antigenic peptides in the context of the class I major histocompatibility complex (MHC). The CTL escape mutations occurring HIV-1 infection has been well documented (Leslie et al., 2004; Gao et al., 2005). In many cases, the replication fitness of the escape mutants is lower than that of the parental viruses (Leslie et al., 2004). Since the CTLs recognize the target viral peptides presented by the MHC on the surface of the virus-infected cells, the efficiency of the CTL response closely depends on the host MHC (human leukocyte antigen, HLA, in human) alleles. Numerous studies show that some HLA alleles, such as HLA-B^*^27 and HLA-B^*^57, have stronger protective effects than that of other HLA alleles (Carrington and O'Brien, 2003; Gao et al., 2005; Goulder and Watkins, 2008). HIV-1-specific CD8+ T-cell responses restricted by these alleles provide a probable mechanism for the protection of HIV-1 infected carriers from disease progression. Notably, HLA-B^*^27 and HLA-B^*^57 targeting epitopes are located in the viral CA protein (mapped in Figure 2). The major escape variant from HLA-B^*^27, R132K, has been altered CypA dependency for viral infection (Schneidewind et al., 2007). The three major highly conserved epitopes of HLA-B^*^57, IW9, KF11, and TW10 are located distant regions in CA. In these epitopes, IW9 and KF11 locate the region that has been identified as the CA NTD-NTD interacting surface. Escape mutations in those regions may confer a structural disadvantage on the virus, one which impairs infectivity (Llano et al., 2009; Pornillos et al., 2009; Brennan et al., 2012). TW10 locates the outer surface of the CA core structure, and Brockman et al. (2007) showed that escape mutants in this region also alter the CypA dependency for its replication. In addition, Battivelli et al. (2011) reported that the mutations in TW10 increase the sensitivity to the potent host cellular restriction factor TRIM5alpha. Thus, escape mutations in CA often induce impairment of viral replication as a result of a failure of interactions with viral or host factors. More generally, it could be said that the interaction between CA and viral or host proteins limits the evolutionary flexibility of the virus. The CTL efficiency is not solely depending on the choice of the epitopes. Even though various factors impact on effective CTL responses such as presentation efficiency of the epitopes, the low evolutional flexibility of the target epitope regions could be important factor for selecting effective CTL responses.

## Perspective

For optimization of anti-HIV-1 therapies, much remains to be studied about the function of each HIV-1 encoded protein and its interacting partner. It is widely known that escape mutants arising under selective pressure from the host immune response or anti-viral therapies often lose the fitness for replication. Recent advances in structural and genomic analyses expand our understanding of viral and host protein interactions. When the host immune response targets such a multi-functional protein, like CA, the possibilities for generating a successful escape mutation may be limited. In fact, two major protective CTL alleles target a region that is functionally important for HIV-1 replication. It can be considered that the multi-functionality of the protein limits the “robustness” of the HIV-1 as a living organism. From this point of view, further functional analysis of the CA protein, as well as CA-host protein interactions, may contribute to establish better therapies for HIV-1.

### Conflict of interest statement

The authors declare that the research was conducted in the absence of any commercial or financial relationships that could be construed as a potential conflict of interest.
